# Activation of Inflammatory and Coagulation Pathways in Patients with Graft-Versus-Host Disease

**DOI:** 10.3390/cells15181690

**Published:** 2026-09-17

**Authors:** Emilija Živković, Milena Todorović Balint, Tijana Subotički, Miloš Diklić, Dragoslava Đikić, Milica Vukotić, Nevena Bešević, Andrej Pešić, Vladan P. Čokić, Olivera Mitrović Ajtić

**Affiliations:** 1 Institute for Medical Research, National Institute of Republic of Serbia, University of Belgrade, Dr Subotica 4, 11129 Belgrade, Serbia; emilija.zivkovic@imi.bg.ac.rs (E.Ž.); vl@imi.bg.ac.rs (V.P.Č.); 2 Clinic for Hematology, University Clinical Center of Serbia, Dr Koste Todorovića 2, 11000 Belgrade, Serbia; bb.lena@gmail.com (M.T.B.);; 3 Faculty of Medicine, University of Belgrade, Dr Subotića starijeg 8, 11000 Belgrade, Serbia

**Keywords:** graft-versus-host disease (GvHD), thromboinflammation, neutrophil extracellular traps (NETs), platelet–monocyte aggregates, inflammatory signaling pathways

## Abstract

Graft-versus-host disease (GvHD) remains a major complication in patients with hematological malignancies undergoing hematopoietic stem cell transplantation, driven by interconnected neutrophil activation, endothelial dysfunction, inflammation, and dysregulated coagulation. However, characterization of these pathways in GvHD patients remains limited. We used ELISA and flow cytometry to quantify biomarkers of neutrophil extracellular traps (NETs), cytokines, endothelial activation, fibrinolysis, coagulation, and immune cell subsets in peripheral blood of GvHD patients, and Western blot to assess NF-κB, STAT3, and p38 MAPK signaling. GvHD patients showed a thromboinflammatory profile, with elevated NET markers (MPO, cell-free DNA), increased cytokines (IL-1β, IL-8, MCP-1, TNF-α), and amplified platelet activation (P-selectin). Coagulation and fibrinolytic activity were enhanced, reflected by increased tissue factor (TF) and urokinase plasminogen activator (uPA), alongside higher frequencies of TF-expressing monocytes and platelet–monocyte aggregates. In vitro, TNF-α activated NF-κB, STAT3, and p38 MAPK signaling and increased ICAM-1- and VCAM-1-positive mononuclear cells, indicating a proinflammatory, adhesive phenotype, while transendothelial migration and nitric oxide levels were reduced. Leukemia patients with GvHD thus show a coordinated thromboinflammatory response involving NET activation, platelet–monocyte interactions, inflammatory signaling, endothelial dysfunction, and coagulation–fibrinolytic dysregulation, providing mechanistic insight into GvHD pathophysiology and highlighting circulating biomarkers as candidates for early detection, monitoring, and therapeutic intervention.

## 1. Introduction

Graft-versus-host disease (GvHD) is a critical complication following hematopoietic stem cell transplantation and is the major cause of morbidity and mortality associated with transplantation. The mechanism behind GvHD is extremely complex, involving donor T-cell activation, conditioning-induced tissue damage, excess cytokine production, endothelial activation, and vascular dysfunction [1]. While adaptive immunity has traditionally been viewed as playing an important role in the disease process, there is increasing evidence that innate immune cells, such as neutrophils, monocytes, and platelets, are vital in promoting inflammation and coagulation and facilitating the development of thromboinflammation in GvHD patients [2].

The formation of neutrophil extracellular traps (NETs) represents another important feature of inflammatory responses, characterized by the formation of web-like structures composed of decondensed chromatin associated with histones and granular proteins, including myeloperoxidase (MPO) [3]. While NET formation is an important antimicrobial defense mechanism, excessive NET release has been implicated in endothelial injury, activation of coagulation pathways, and the transmission of thromboinflammation [4]. Enhanced NET formation has been described in numerous inflammatory and autoimmune disorders [5]; however, its contribution to GvHD pathophysiology remains incompletely understood. In parallel, dysregulation of coagulation and fibrinolytic pathways has emerged as an important feature of GvHD, where inflammatory mediators may induce tissue factor (TF) expression, platelet activation, and endothelial dysfunction, thereby establishing a prothrombotic microenvironment [6].

Interactions between monocytes and platelets represent important links between inflammation and coagulation in patients with GvHD. Activated monocytes are able to express TF and directly initiate the extrinsic coagulation cascade, while platelet–leukocyte interactions further amplify inflammatory signaling and vascular injury [7]. Altered expressions of adhesion molecules, including intercellular adhesion molecule-1 (ICAM-1) and vascular cell adhesion molecule-1 (VCAM-1), reflect immune cell activation and may contribute to leukocyte trafficking and inflammatory tissue injury in GvHD [8,9]. Accordingly, endothelial activation and vascular dysfunction are gradually recognized as central pathogenic events associated with adverse clinical outcomes in GvHD patients [10].

Hypoxia and the hypoxia-inducible factor-1 alpha (HIF-1α) signaling pathway have emerged as additional mechanisms contributing to the maintenance of inflammatory responses in GvHD [11]. HIF-1α regulates the expression of numerous genes involved in inflammation, cellular adhesion, metabolic adaptation, and immune cell activation. Importantly, proinflammatory cytokines such as tumor necrosis factor-alpha (TNF-α) and interleukin-1 beta (IL-1β) can induce HIF-1α stabilization even under normoxic conditions, thereby enhancing inflammatory signaling independently of oxygen deprivation [12]. Furthermore, activation of key inflammatory pathways, including nuclear factor kappa B (NF-κB), signal transducer and activator of transcription 3 (STAT3), and p38 mitogen-activated protein kinase (p38 MAPK), has been associated with cytokine production, immune cell activation, endothelial dysfunction, and progression of inflammatory tissue injury [13].

Although previous studies have demonstrated associations between inflammation, coagulation abnormalities, and vascular dysfunction in GvHD, the integrated contribution of NET formation, thromboinflammation, hypoxia-associated signaling, and inflammatory pathway activation remains incompletely understood. Therefore, the aim of the present study was to investigate the systemic inflammatory and procoagulant profile of patients with GvHD, with a particular focus on NET formation, fibrinolytic activity, phenotypic alterations of neutrophils and monocytes, platelet and endothelial activation, as well as hypoxia-related signaling and activation of the NF-κB, STAT3, and p38 MAPK inflammatory pathways. A better understanding of these interconnected molecular mechanisms may provide deeper insights into the pathophysiology of GvHD and identify potential therapeutic targets for the modulation of thromboinflammatory responses and vascular injury in patients with GvHD.

## 2. Materials and Methods

### 2.1. Patients’ Characteristics

In the study, 29 diagnosed GvHD patients were prospectively recruited from the Clinic of Hematology, University Clinical Center of Serbia, Belgrade, Serbia. The mean patients’ age was 35.54 ± 8 years (range, 2–62 years); 12 (41.4%) were women. The patients included in this study initially had diagnosis of acute lymphoblastic leukemia (ALL 10; 34.4%), acute myeloid leukemia (AML 7; 24.2%), Hodgkin lymphoma (HL 6; 20.7%), myelodysplastic syndrome (MDS 3; 10.3 %), non-Hodgkin lymphoma (NHL 1; 3.4%), primary myelofibrosis (1; 3.4%), and aplastic anemia (1; 3.4%), while only 7 patients relapsed (24.2% (4 HL and 3 AML patients)). The average event-free survival (EFS) was 53.52 ± 45 months (range, 11–229 months), while the average time between stem cell transplantation and the onset of GvHD was 8.5 ± 7 months (range, 1–38 months). Venous and arterial thrombosis were diagnosed in 17.2% (*n* = 5) of GvHD patients. We also examined 18 healthy controls involving 10 females (age 41.9 ± 10 years) and 8 males (age 43.1 ± 8.7 years). Informed consent was obtained from all the participants included in the study, approved by the local Ethical Committee. Demographic and clinical characteristics of the GvHD cohorts are presented in Supplementary Materials Table S1.

### 2.2. Isolation and Preparation of Cells from Peripheral Blood

Peripheral blood (30 mL) of GvHD patients was used to isolate mononuclear cells (MNC), endothelial cells, neutrophils and platelets. Blood collection was done using BD Vacutainer K2E EDTA (BD Switzerland Sarl, Eysins, Switzerland). Cells isolation was performed using Lymphocyte Cell Separation Media (Capricorn Scientific, Ebsdorfergrund, Germany). Circulating endothelial cells were isolated from whole blood by using BD FACS lysing solution (BD 349202, Franklin Lakes, NJ, USA). Proteins were isolated from whole cell pellets of MNCs for performing Western blot analysis. MNCs, endothelial cells, neutrophils and platelets were used for quantifying CD markers on the cell surface.

### 2.3. Determination of NET Quantity from the Plasma of the GvHD Patients and Healthy Controls

The MPO activity and the concentrations of cell-free DNA (cfDNA) were determined in the plasma isolated from the blood samples of patients with GvHD (1–10 from Supplementary Materials Table S1) and control volunteers (10). In the case of NETs, blood samples were taken in tubes pre-treated with EDTA. After separation of plasma by centrifugation, it was analyzed for the following NET biomarkers: cfDNA concentration (Abcam, Cambridge, UK) and MPO activity (Elabscience, Houston, TX, USA). The fluorescence was recorded using the PerkinElmer Wallac 1420 Victor2 spectrophotometer (PerkinElmer, Buckinghamshire, UK) and the absorbance was determined on an ELISA microplate reader (RT-6100, Raito, Shenzhen, China).

### 2.4. Tissue-Type Plasminogen Activator and Urokinase Activity in GvHD Patients

The fibrinolytic capacity of the sample was assessed by estimating the levels of tissue-type plasminogen activator (tPA) and urokinase in patients with GvHD (1–10 from Supplementary Materials Table S1). The Human tPA Activity Assay Kit (Cat. No. ab 108905; Abcam, Waltham, MA, USA) was used for quantifying the level of human tPA activity in the plasma sample from patients with GvHD. The plasmin level was estimated by using the highly specific plasmin substrate which releases para-nitroaniline (pNA) chromophore, and the level of pNA was detected based on the absorbance at 405 nm. Urokinase activity was determined by using the Urokinase Activity Fluorometric Assay Kit (Sigma-Aldrich, Burlington, MA, USA). The fluorescence was generated after the enzymatic reaction, which is proportional to the urokinase activity (ex = 350/nm = 450 nm).

### 2.5. ELISA Assay of Inflammatory and Coagulation Parameters in GvHD Patients

The quantitation of proinflammatory mediators such as IL-1β (patients 1–28), TNF-α, MCP-1, IL-8 (1–16, 28, 29), HIF1α (21–23 from Supplementary Materials Table S1), as well as coagulation mediators such as FVIII (patients 1–28), sP-selectin, thrombin, and TF (1–16, 28, 29 from Supplementary Materials Table S1), in the plasma of the GvHD patient and the healthy donor was done using ELISA kits (Elabscience) following manufacturer’s recommendations. All the plasma samples were tested in triplicate, and values were calculated from the standard curve that was generated by recombinant standards and given as average values in appropriate units for each of the tested cytokines and coagulation parameters.

### 2.6. Flow Cytometry

Cell surface marker expression on the cell surface was analyzed by flow cytometry of peripheral blood (Supplementary Materials Table S2). All types of investigated cells (neutrophils, monocytes, platelets, circulating endothelial cells, platelet–monocyte aggregates) were fixed in 4% paraformaldehyde and stained with fluorescein isothiocyanate (FITC)-, phycoerythrin (PE)-, allophycocyanin (APC)-, or peridinin chlorophyll protein complex/cyanine 5.5 (PerCP/Cy5.5)-conjugated antibodies directed against human CD16-FITC (225367, Elabscience), CD142-APC (365206, BioLegend, San Diego, CA, USA), and CD14-PE (FW1347, Elabscience) for monocytes, CD31-PE (303106, BioLegend) for circulating endothelial cells, CD11b-PE (225358, Elabscience) for neutrophils (patients 1–5 from Supplementary Materials Table S1), CD61-PE (225359, Elabscience) for platelets (patients 3–6 and 15), CD62P-FITC (304904, BioLegend) and CD63-PE (353004, BioLegend) for activated platelets (patients 16–20), and CD45-FITC (304054, BioLegend) and CD41-PerCP/Cy5.5 (303720, BioLegend) for platelet–monocyte aggregates (patients 6 and 15–18 from Supplementary Materials Table S1). The gating strategy was based on sequential exclusion of debris and noncellular events according to FSC/SSC characteristics, followed by identification of the relevant cell populations based on the expression of specific CD markers. Neutrophils were identified within the granulocyte population and further subdivided according to CD16 and CD11b expression into CD16^+^CD11b^−^, CD16^−^CD11b^+^ and CD16^+^CD11b^+^ populations. Platelet–monocyte aggregates were identified by sequential gating of CD45^+^CD14^+^ monocytes followed by assessment of CD41 expression. Events positive for CD45 and CD14 and simultaneously positive for CD41 were classified as platelet–monocyte aggregates. CD41^+^ events lacking CD45/CD14 expression were not included in the platelet–monocyte aggregate population. Labeled cells were analyzed using BD FACS Calibur (BD Bioscience, Franklin Lakes, NJ, USA). For each sample, at least 10,000 events were recorded. Flow cytometry data were analyzed using FlowJo software version 10.8.1 (BD, Ashland, OR, USA).

### 2.7. Endothelial Cell Co-Culture with Mononuclear Cells in Boyden Chamber 

HMEC-1 (human microvascular endothelial cells-1) is an endothelial cell line derived from the microvascular endothelium of foreskin tissue in a male patient. The HMEC-1 cells were obtained from ATCC (TIB-180, Manassas, VA, USA). HMEC-1 cells were cultured in microvascular endothelial cell growth medium kit (enhanced) (PELOBiotech, Planegg, Germany) supplemented with 10% FBS, 1% glutamine, and 1% penicillin/streptomycin. HMEC-1 cells were incubated at 37 °C and 5% CO_2_ in culture and kept within a cell concentration range between 8 × 10^5^ and 1 × 10^6^ cells/mL, cells were washed in PBS, and the medium was replaced every two days. A 24-well cell migration assay kit (Cat. No. CBA-102; Cell Biolabs, Inc., San Diego, CA, USA) was used to assess migration of MNCs from GvHD patients (11, 19, 24, 26, 28, 29 from Supplementary Materials Table S1) and control individuals across the endothelial barrier to the lower chamber. HMEC-1 cells (5 × 10^5^ cells/well) were seeded as a confluent monolayer on the 4 μm pore membrane, in the Boyden chamber’s upper compartment with or without TNF-α treatment (Miltenyi Biotec, Bergisch Gladbach, Germany). After 24 h, mononuclear cells (MNC, 2 × 10^5^ cells/well) were added and co-cultured with a monolayer of HMEC-1 and incubated for 24 h. The migratory cells were labeled with CyQuant GR dye and fluorescence measurement was performed with Wallac 1420 Victor^2^ Microplate Reader (PerkinElmer, Waltham, MA, USA) at 480/520 nm. 

### 2.8. Assessment of ICAM-1 and VCAM-1 Expression Under Normoxic and Hypoxic Conditions

The cultures of Granulocytes and MNCs from GvHD patients (21–23 from Supplementary Materials Table S1) were conducted in a cell growth medium kit improved (PELOBiotech) with 10% FBS, 1% glutamine and 1% penicillin/streptomycin. The samples were incubated at 37 °C under normoxia and hypoxia (2% O_2_) conditions and the seeding concentration was 7 × 10^5^ cells/mL. The cells were exposed to TNF-α (5 ng/mL, Miltenyi Biotec) for 24 h. The cells (granulocytes and MNCs (2 × 10^4^ cells per slide)) were centrifuged and fixed on Superfrost™ microscope slides with methanol. Non-specific staining due to peroxidase was blocked by treatment of samples with 3 % hydrogen peroxide. The samples were further treated with the respective primary antibodies ICAM-1 (Cat.No. E-AB-67682, Elabscience) and VCAM-1 (Cat. No. E-AB-70284, Elabscience) overnight at +4 °C. Staining was done by the streptavidin–biotin method (LSAB+/HRP Kit, DAKO, Glostrup, Denmark). The DAKO Liquid DAB^+^ Substrate/Chromogen System (DAKO) was used for visualization of immunoreactivity, whereas contrast for unstained nuclei was provided by Mayer’s hematoxylin (Merck, Whitehouse Station, NY, USA). As a negative control, the samples incubated with PBS without a primary antibody were used. Five random microscopic fields per section with an average number of ICAM-1 and VCAM-1 positive cells were taken and processed. The imaging was carried out under a light microscope (Olympus AX70, Hamburg, Germany) and the counting of immunoreactive cells was done using the Analysis Pro 3.1 software (Olympus Soft Imaging Solutions GmbH, Münster, Germany). The magnification was 40×.

### 2.9. Western Blot

The whole cell extract was obtained from cell pellets of MNCs by extraction in RIPA buffer (1  mM EDTA, 50  mM Tris-HCl pH 7.5, 0.1% SDS, 150 mM NaCl, 1% NP40, 1% Sodium deoxycholate, protease inhibitor cocktail). Homogenates were separated into polyacrylamide gels and transferred onto polyvinylidene difluoride membranes (GE Healthcare, Chicago, IL, USA). Signals were detected, from patients with GvHD (1, 4, 5, 7, 8, 10–20, 25, 28 from Supplementary Materials Table S1), using antibodies directed against phospho-NFκB (Ser536, Cat. No. 3031, Cell Signaling, Danvers, MA, USA), total NFκB (Cat. No. E-AB-22066, Elabscience), phospho-p38 (Tyr 180/Tyr 182, Cat. No. E-AB-21027, Elabscience), total p38 (Cat. No. E-AB-66279, Elabscience), STAT3 (Cat. No. 9132, Cell Signaling) and pSTAT3 (Tyr705, Cat. No. 9131S, Cell Signaling) and incubated overnight at 4 °C (Supplementary Materials Table S2). Secondary antibodies conjugated to horseradish peroxidase (Elabscience) were detected using an enhanced chemiluminescence detection system (Bio-Rad Laboratories, Hercules, CA, USA). Protein bands were visualized using ChemiDoc ™ Imaging System (Bio-Rad Laboratories) and quantified using Image Lab software (Version 6.0.0.25, Bio-Rad Laboratories). 

### 2.10. Ozone-Based Chemiluminescent Determination of Nitrite in GvHD Plasma

Plasma nitrite (NO2^−^) determination involved deproteinizing plasma samples using cold 96% ethanol (500 µL of plasma/1 mL of ethanol) and then incubating the samples for 30 min at 20 °C. Thereafter, the samples were centrifuged for 15 min at 4 °C at 13,000 rpm and the supernatants were collected into new tubes. Nitrite analysis was done using Sievers Model 280 Nitric Oxide Analyzer (Sievers, Boulder, CO, USA) as previously described [14]. Plasma nitrite was first converted to nitric oxide (NO) through chemical reduction using acidified potassium iodide (PI) solution containing 4 mL glacial acetic acid, 1 mL distilled water, and 50 mg PI. Therefore, plasma nitrite concentrations were determined by ozone-based chemiluminescence following quantitative reduction of nitrite to NO from patients with GvHD (1–3, 8–14, 16–24, and 26 from Supplementary Materials Table S1).

### 2.11. Statistical Analysis

The normality of the data was assessed by applying the Shapiro–Wilk and Kolmogorov–Smirnov tests. The values are represented in terms of mean ± standard deviation. Comparisons between groups were done through paired or unpaired Student’s *t*-test using GraphPad Prism software version 8.0.0 for Windows (GraphPad Software, San Diego, CA, USA). If the data had failed to show normality, the non-parametric Mann–Whitney U test was used. The correlations were assessed by Spearman’s or Pearson’s correlation coefficients depending on the normality of data distribution. A two-sided P-value of less than 0.05 was considered significant.

## 3. Results

### 3.1. Thromboinflammatory Alterations in Graft-Versus-Host Disease

**Figure 1:**
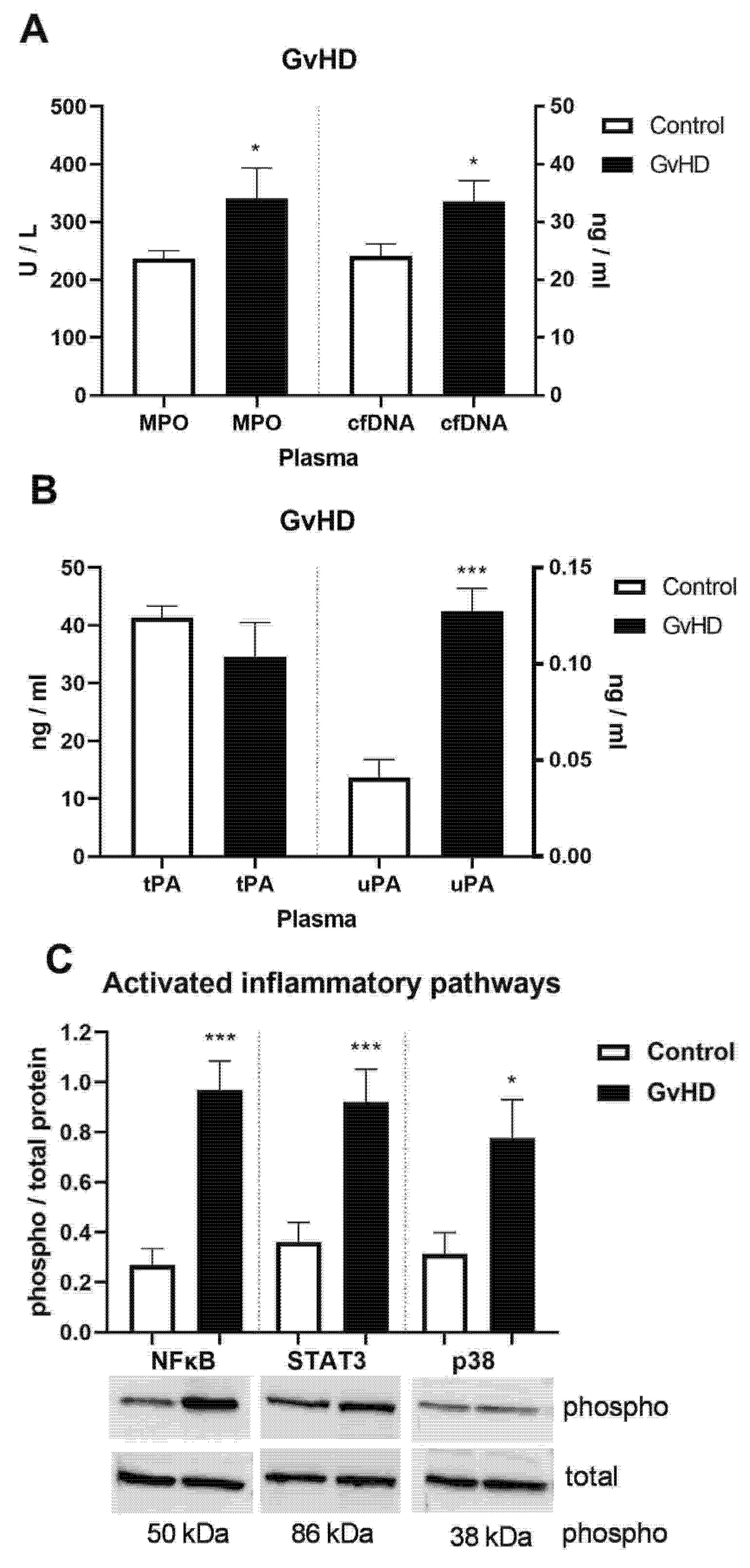
NET formation, fibrinolytic activity, and activation of inflammatory signaling pathways in graft-versus-host disease. Level of biomarkers of neutrophil extracellular traps (NETs) in plasma of peripheral blood of: (**A**) myeloperoxidase (MPO) and cell-free DNA (cfDNA) of healthy volunteers (Control, *n* = 18 for MPO and *n* = 8 for cfDNA) and patients with graft-versus-host disease (GvHD, *n* = 10). (**B**) Level of fibrinolytic factor tissue plasminogen activator (tPA) and urokinase plasminogen activator (uPA) of healthy volunteers (Control, *n* = 5) and patients with GvHD (*n* = 10). (**C**) Peripheral blood mononuclear cells (MNCs) were isolated from patients with GvHD (*n* = 18) and activation of NFκB, STAT3 and p38 MAPK signaling pathways were examined in comparison to healthy volunteers (Control, *n* = 10). Representative immunoblots are shown for each signaling pathway and correspond to one independent experiment. Quantitative bar graphs represent the mean ± SEM of densitometric analyses from independent biological experiments after normalization of phosphorylated proteins to the corresponding total protein bands. * *p* < 0.05, *** *p* < 0.001 vs. Control. Unpaired (Control vs. GvHD) two-tailed Student’s *t*-tests were used for normally distributed data (**A**–**C**), whereas the Mann–Whitney U test was used for non-parametric data (**C**, for STAT3).

To evaluate whether neutrophil activation contributes to the inflammatory profile of GvHD, we first quantified circulating biomarkers of NETs in plasma (Figure 1A). MPO, a marker of neutrophil degranulation and NET release, was significantly increased in patients with GvHD (*p* < 0.05) compared with healthy controls, and further increased in acute compared with chronic GvHD (*p* = 0.0063, unpaired two-tailed Student’s *t*-test). Concentrations of cfDNA, a hallmark component of NET structures, were also markedly elevated (*p* < 0.05, Figure 1A). We next assessed fibrinolytic activity (Figure 1B): plasma tPA activity tended to be lower in GvHD, whereas uPA was significantly increased (*p* < 0.001), suggesting dysregulated fibrinolysis. Western blot analysis of MNCs demonstrated marked activation of inflammatory signaling pathways in GvHD, with significantly increased phosphorylation of NF-κB (*p* < 0.001), STAT3 (*p* < 0.001), and p38 MAPK (*p* < 0.05, Figure 1C). STAT3 levels correlated positively with IL-8 (Spearman, ρ = 0.587, 95% CI 0.001–0.873, *p* = 0.049, *n* = 12) and thrombin (ρ = 0.642, 95% CI 0.089–0.893, *p* = 0.028, *n* = 12). Together, these data demonstrate coordinated activation of NET formation, dysregulated fibrinolysis, and proinflammatory intracellular signaling, consistent with a systemic thromboinflammatory state in GvHD.

### 3.2. Systemic Inflammatory and Coagulation Markers in Patients with GvHD

**Figure 2:**
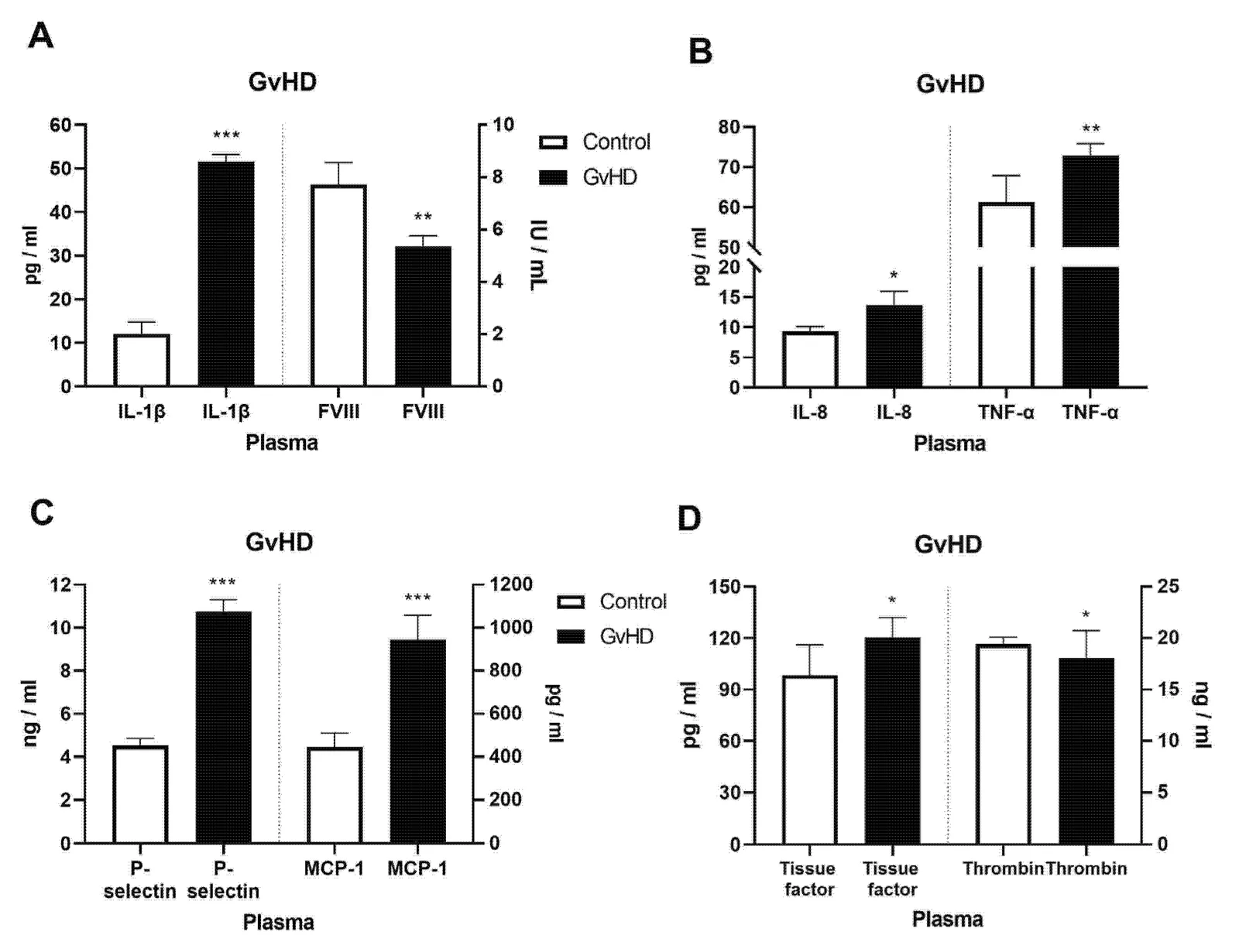
Expression of inflammatory cytokines and coagulation factors in patients with graft-versus-host disease. Plasma levels of (**A**) interleukin-1β (IL-1β) and factor VIII (FVIII, *n* = 28); (**B**) interleukin-8 (IL-8) and tumor necrosis factor-alpha (TNF-α, *n* = 18); (**C**) P-selectin and monocyte chemoattractant protein-1 (MCP-1, *n* = 18); and (**D**) tissue factor and thrombin (*n* = 18) were determined in healthy volunteers (Control, *n* = 18) and patients with graft-versus-host disease (GvHD). Values are mean ± SEM, while Mann–Whitney U test was used for non-parametric data. * *p* < 0.05, ** *p* < 0.01, *** *p* < 0.001 vs. Control.

To further characterize this hemostatic profile, we quantified circulating cytokines and coagulation-related biomarkers (Figure 2). IL-1β (Figure 2A) was markedly increased in GvHD (*p* < 0.001), while plasma FVIII was significantly decreased (*p* < 0.01) and, within the GvHD group, higher in chronic than acute disease (*p* = 0.0202, Mann–Whitney U). IL-8 (*p* < 0.05) and TNF-α (*p* < 0.001, Figure 2B) were also significantly elevated, with TNF-α showing the strongest increase. FVIII correlated positively with IL-8 (Spearman, ρ = 0.567, 95% CI 0.121–0.822, *p* = 0.014, *n* = 18). Markers of endothelial and platelet activation (Figure 2C) were similarly upregulated: P-selectin was nearly doubled in GvHD (*p* < 0.001) and correlated positively with overall survival (Spearman, ρ = 0.533, 95% CI 0.035–0.819, *p* = 0.033, *n* = 16), and MCP-1 was markedly elevated (*p* < 0.001). Among coagulation mediators (Figure 2D), TF was significantly increased (*p* < 0.05), whereas thrombin was significantly decreased (*p* < 0.05), possibly reflecting increased consumption or altered coagulation regulation. 

### 3.3. Neutrophil and Monocyte Subsets in GvHD

**Figure 3:**
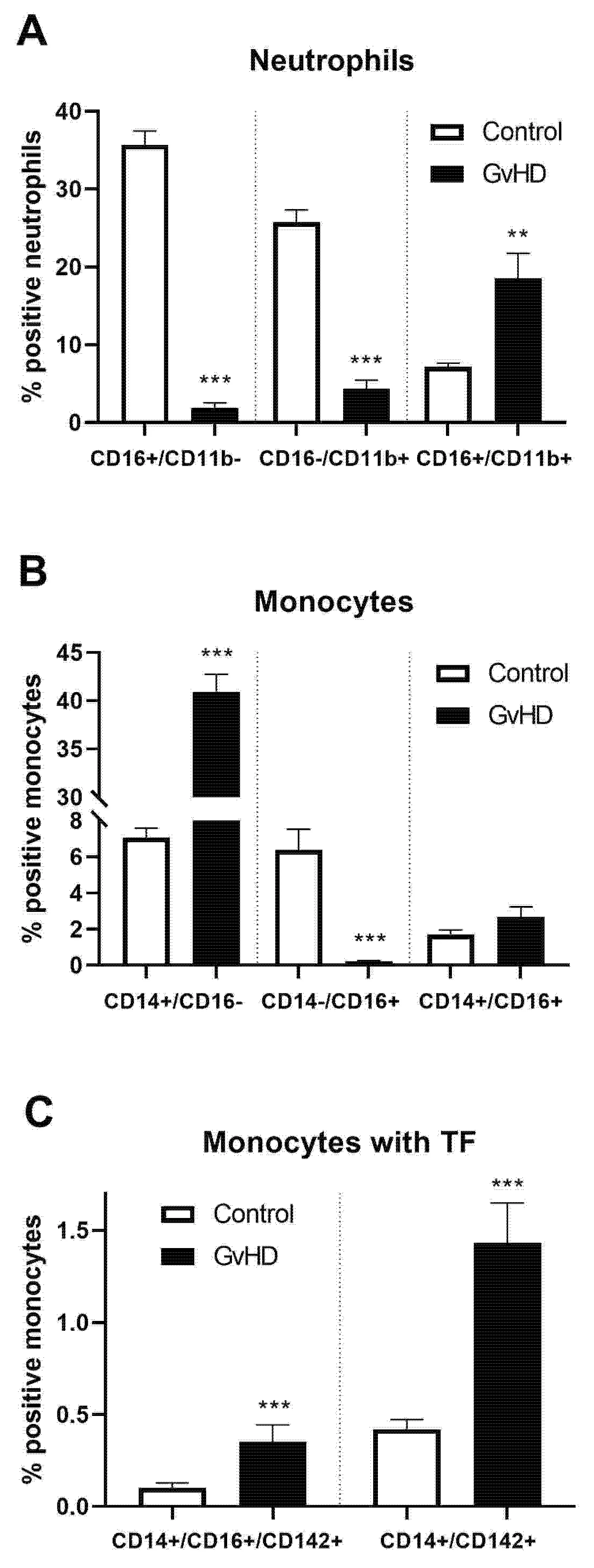
Immune cell populations and tissue factor expression in patients with graft-versus-host disease. Using flow cytometry, we determined the frequency of (**A**) CD16^+^CD11b^−^, CD16^−^CD11b^+^, and CD16^+^CD11b^+^ neutrophils; (**B**) CD14^+^CD16^−^, CD14^−^CD16^+^, and CD14^+^CD16^+^ monocytes; and (**C**) tissue factor (CD142) expression within the CD14^+^CD16^+^ monocyte population (left pair of columns) and CD14^+^ monocytes (right pair of columns) in patients with graft-versus-host disease (GvHD, *n* = 5) and healthy volunteers (Control, *n* = 5). Representative flow cytometry gating strategies are shown in Supplementary Materials Figure S1. Values are mean ± SEM, while Mann–Whitney U test was used for non-parametric data. ** *p* < 0.01, *** *p* < 0.001 vs. corresponding Control.

Flow cytometric profiling revealed marked shifts in circulating neutrophil and monocyte populations. Among neutrophil subsets (Figure 3A), CD16^+^/CD11b^−^ and CD16^−^/CD11b^+^ neutrophils were both significantly reduced (*p* < 0.001) in GvHD, while the activated CD16^+^/CD11b^+^ double-positive subset was significantly increased (*p* < 0.01). Monocyte profiling (Figure 3B) showed a several-fold increase in classical CD14^+^/CD16^−^ monocytes (*p* < 0.001), near-absence of the non-classical CD14^−^/CD16^+^ subset (p < 0.001), and a mild, non-significant increase in intermediate CD14^+^/CD16^+^ monocytes. TF expression on monocytes (Figure 3C) was likewise increased: CD14^+^/CD16^+^/CD142^+^ and CD14^+^/CD142^+^ populations were both significantly elevated (*p* < 0.001) in GvHD.

### 3.4. Circulating Thromboinflammatory Cell Populations in GvHD

**Figure 4:**
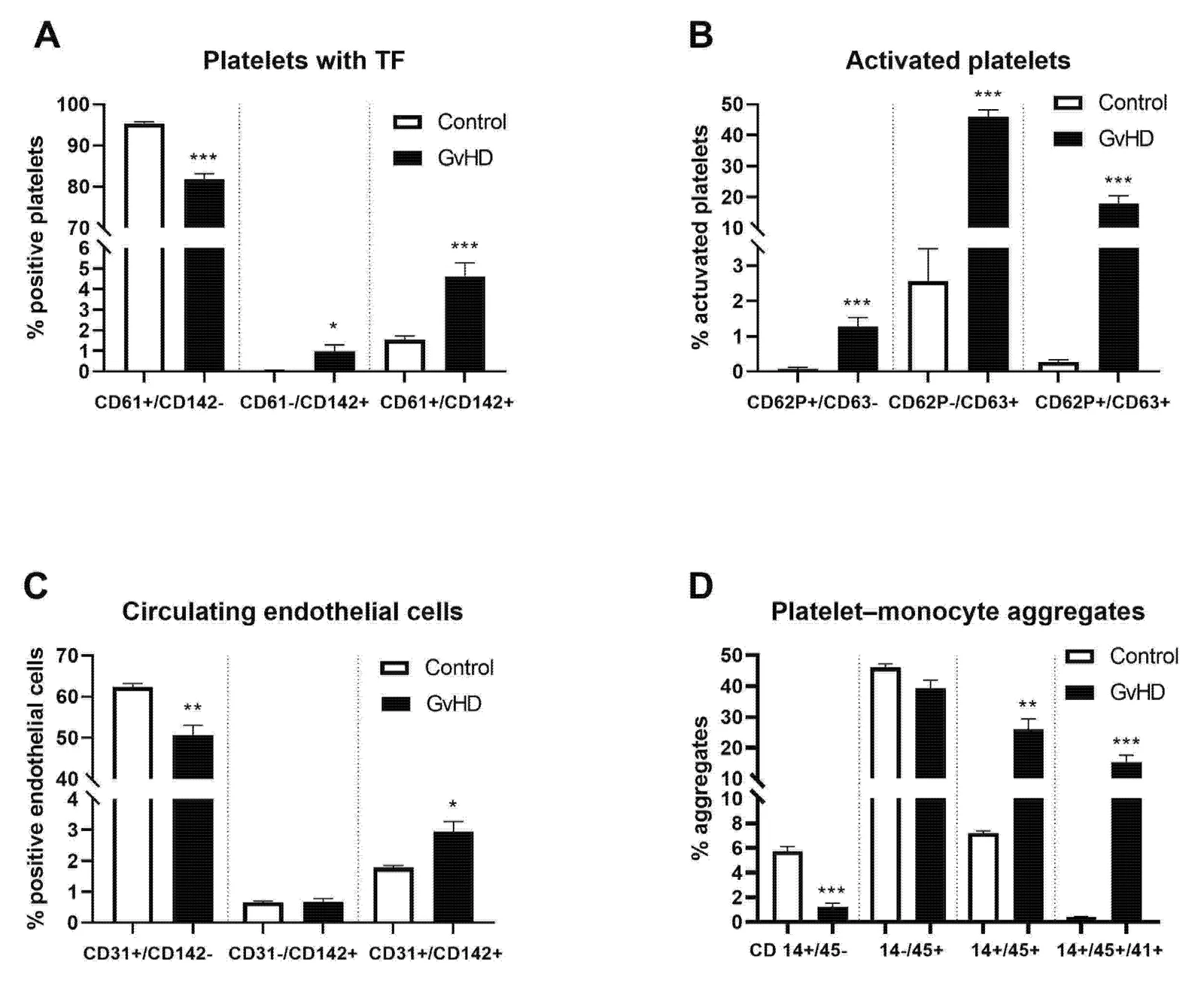
Circulating coagulation- and endothelial-related cell populations in patients with graft-versus-host disease. Using flow cytometry, we determined the frequency of (**A**) CD61^+^CD142^−^ platelets, CD61^−^CD142^+^ tissue factor-positive cells, and CD61^+^CD142^+^ tissue factor-positive platelets; (**B**) activated platelets (CD62P^+^ and CD63^+^); (**C**) CD31^+^ endothelial cells and CD31^+^CD142^+^ tissue factor-positive endothelial cells; and (**D**) platelet–monocyte aggregates, identified as CD41^+^ events within the CD14^+^CD45^+^ monocyte population, in patients with graft-versus-host disease (GvHD, *n* = 5) and healthy volunteers (Control, *n* = 5). Representative flow cytometry gating strategies are shown in Supplementary Materials Figure S2. Values are mean ± SEM, while Mann–Whitney U test was used for non-parametric data. * *p* < 0.05, ** *p* < 0.01, *** *p* < 0.001 vs. the corresponding Control.

Circulating platelet, endothelial, and platelet–monocyte populations showed parallel disturbances. CD61^+^/CD142^−^ platelets were significantly reduced (*p* < 0.001), while CD61^−^/CD142^+^ (*p* < 0.05) and CD61^+^/CD142^+^ (*p* < 0.001) subsets were significantly increased (Figure 4A), the latter correlated positively with tPA (Pearson, r = 0.952, r^2^ = 0.907, 95% CI −0.104–0.999, *p* = 0.049, *n* = 5). Platelet activation markers (Figure 4B)—CD62P^+^/CD63^−^, CD62P^−^/CD63^+^, and CD62P^+^/CD63^+^—were all significantly increased (*p* < 0.001 each), with the CD63^+^ population showing the greatest change. Endothelial cell profiling (Figure 4C) showed a significant decrease in CD31^+^/CD142^−^ cells (*p* < 0.01) and a significant increase in CD31^+^/CD142^+^ cells (*p* < 0.05). Platelet–monocyte aggregates (Figure 4D) followed the same pattern: CD14^+^/CD45^−^ events were significantly reduced (*p* < 0.001), CD14^+^/CD45^+^ aggregates significantly increased (*p* < 0.01), and CD14^+^/CD45^+^/CD41^+^ aggregates showed the most pronounced increase (*p* < 0.001).

### 3.5. Hypoxia-Associated HIF-1α Signaling and Adhesion Molecule Expression in GvHD

**Figure 5:**
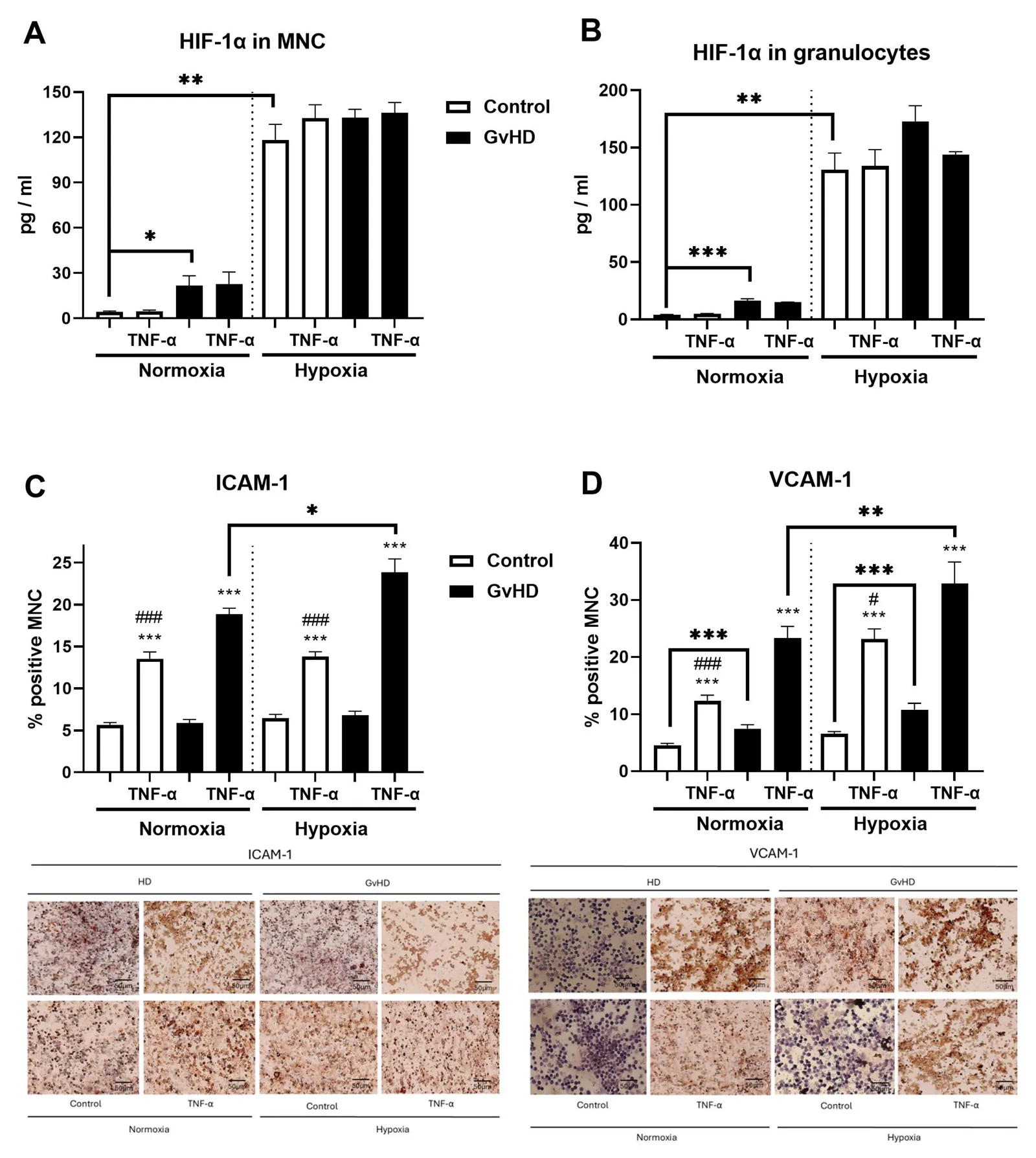
Hypoxia-induced expression of HIF-1α and adhesion molecules in patients with graft-versus-host disease. HIF-1α levels were determined by ELISA in paired (**A**) circulating mononuclear cells (MNC) and (**B**) granulocytes obtained from the same patients with graft-versus-host disease (GvHD, *n* = 3) and healthy volunteers (Control, *n* = 4). The expression of (**C**) intercellular adhesion molecule-1 (ICAM-1) and (**D**) vascular cell adhesion molecule-1 (VCAM-1) was evaluated by immunocytochemistry in MNCs cultured under normoxic or hypoxic (2% O_2_) conditions for 24 h following pretreatment with tumor necrosis factor-alpha (TNF-α, 5 ng/mL) in the same patients and Controls. Representative immunocytochemistry images are shown alongside panels (**C**,**D**). Values are mean ± SEM. * *p* < 0.05, ** *p* < 0.01, *** *p* < 0.001 vs. paired non-treated MNCs or as indicated. ^#^ *p* < 0.05, ^###^ *p* < 0.001 vs. corresponding TNF-α treated MNCs of GvHD under same oxygen tension. Paired (same samples) and unpaired (Control vs. GvHD) two-tailed Student’s *t*-tests were used for normally distributed data (**A**–**C**), whereas the Mann–Whitney U test was used for non-parametric data ((**D**), hypoxia).

Hypoxia-associated signaling was assessed in MNCs and granulocytes. Basal HIF-1α was low under normoxia in both groups; TNF-α stimulation produced a modestly but significantly greater increase in GvHD MNCs than controls (*p* < 0.05), and hypoxia alone significantly increased HIF-1α in GvHD MNCs relative to normoxic conditions (*p* < 0.001, Figure 5A). Granulocytes showed a comparable pattern, with significantly greater TNF-α-induced HIF-1α under normoxia in GvHD (*p* < 0.001, Figure 5B); hypoxia produced a robust elevation in both cell types and in both groups. Adhesion molecule expression in MNCs followed a similar trend: TNF-α significantly increased ICAM-1^+^ MNCs in both groups (*p* < 0.001), with a significantly greater GvHD response under both normoxia (*p* < 0.01) and hypoxia (*p* < 0.001), and a further increase under hypoxia versus normoxia within GvHD (*p* < 0.05, Figure 5C). VCAM-1 expression was significantly higher in GvHD MNCs under both normoxia and hypoxia (*p* < 0.001, Figure 5D); TNF-α increased VCAM-1 in controls (*p* < 0.001), but the GvHD response was significantly greater overall (*p* < 0.001), under normoxia (*p* < 0.01), and under hypoxia (*p* < 0.05), with hypoxia further enhancing the TNF-α-induced response in GvHD (*p* < 0.01).

### 3.6. Transendothelial Migration of Mononuclear Cells and Plasma Nitrite Levels in GvHD

**Figure 6:**
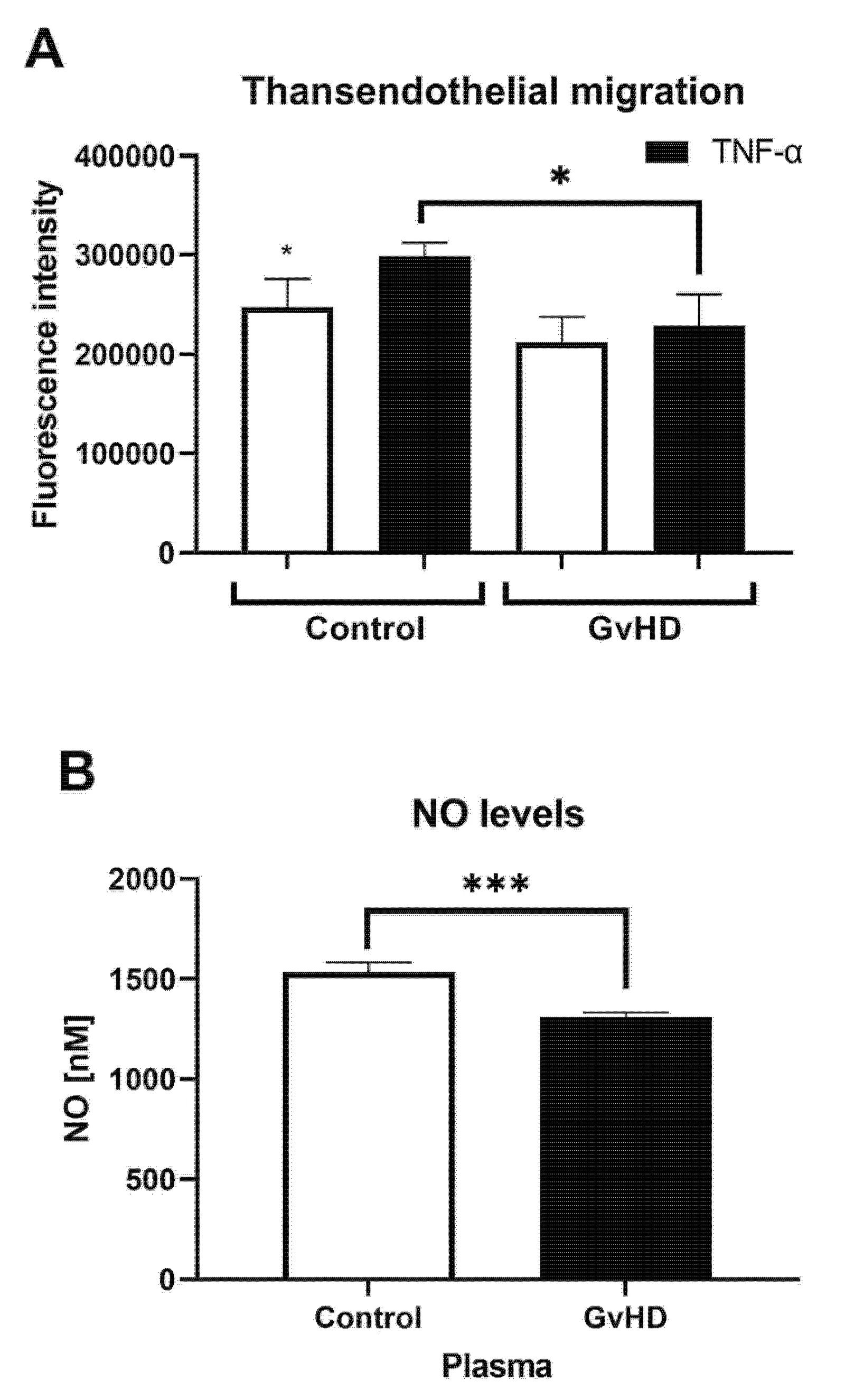
Endothelial cell adhesion and nitric oxide (NO) bioavailability in patients with acute myeloid leukemia and graft-versus-host disease. (**A**) Transendothelial migration assay. Human microvascular endothelial cells (HMEC-1) were cultured on Transwell inserts and stimulated with 5 ng/mL tumor necrosis factor-alpha (TNF-α) for 24 h. Mononuclear cells (MNC), isolated from the peripheral blood of patients with graft-versus-host disease (GvHD) or healthy controls, were added to the upper chamber and allowed to migrate for 24 h. Migrated cells were detached and lysed, and fluorescence intensity was measured as an indicator of transendothelial migration. Data are shown for GvHD (*n* = 6) and volunteers (Control, *n* = 4) under simultaneous TNF-α stimulated or vehicle-treated (non-stimulated) conditions. (**B**) Plasma nitrite levels in peripheral blood of patients with GvHD (*n* = 20) and healthy controls (*n* = 6). White bars represent non-treated cells. Values are mean ± SEM. * *p* < 0.05, *** *p* < 0.001 vs. TNF-α stimulated or as indicated. Unpaired (Control vs. GvHD) two-tailed Student’s *t*-tests were used for normally distributed data ((**A**), Control), whereas the Mann–Whitney U test was used for non-parametric data ((**A**), GvHD; (**B**)).

Functional interaction between MNCs and the vascular endothelium was assessed by transendothelial migration across TNF-α-stimulated HMEC-1 monolayers (Figure 6A). Basal migration was slightly, non-significantly reduced in GvHD MNCs versus controls. TNF-α stimulation produced a non-significant increase in control MNC migration, whereas migration of GvHD MNCs was significantly reduced compared with stimulated controls (*p* < 0.05), indicating impaired transendothelial migratory capacity during acute GvHD. Systemic NO bioavailability, assessed via plasma nitrite (Figure 6B), was significantly lower in GvHD (1310 ± 181 nM) than in controls (1534 ± 210 nM; *p* < 0.001), and correlated positively with GvHD grade (ρ = 0.709, *p* = 0.014, *n* = 9).

## 4. Discussion

We demonstrated that GvHD is associated with marked neutrophil activation, increased NET formation, and amplification of inflammatory signaling, together forming a thromboinflammatory response that is reflected across the plasma, cellular, and vascular compartments. Elevated plasma MPO and cfDNA, together with remodeling of circulating neutrophil populations—depletion of less activated CD16^+^CD11b^−^ and CD16^−^CD11b^+^ subsets alongside expansion of CD16^+^CD11b^+^ proinflammatory neutrophils—indicate a systemic shift toward a highly activated neutrophil compartment [15,16]. Although MPO and cfDNA are individually non-specific for NET formation, their concomitant elevation together with this phenotypic shift is consistent with enhanced NET-related activity in GvHD [17,18,19,20]. Extracellular DNA, histones, and neutrophil-derived proteases released during NETosis promote endothelial activation, platelet adhesion, and induction of tissue factor (TF) [20,21]. A parallel redistribution of monocyte subsets—expansion of classical CD14^+^/CD16^−^ monocytes with reduced non-classical CD14^−^/CD16^+^ monocytes, together with an increased frequency of TF-expressing monocytes—extends this procoagulant signature to a second immune cell compartment, providing a mechanistic link between innate immune activation, endothelial injury, and downstream thrombin generation [22]. These innate immune-driven procoagulant mechanisms resemble those described in pathogen-triggered immunothrombosis, though in GvHD they arise in a sterile, alloimmune context rather than in response to infection—consistent with the broader thromboinflammatory framework used throughout this manuscript. Taken together, these cellular changes establish TF induction as a convergence point linking neutrophil, monocyte, and endothelial activation in GvHD.

This procoagulant cellular environment was accompanied by disturbances in fibrinolytic regulation. Although uPA concentrations were increased, tPA activity remained relatively preserved, indicating compartment-specific dysregulation rather than globally enhanced fibrinolysis. Since uPA primarily drives pericellular proteolysis and inflammatory cell migration whereas tPA governs intravascular fibrin degradation, this imbalance may favor persistence of intravascular fibrin despite increased tissue proteolysis—linking immune activation to the heightened thrombotic risk observed in GvHD [23,24]. Reduced circulating FVIII and thrombin, set against increased TF expression, may reflect increased consumption or altered coagulation regulation rather than reduced procoagulant drive, underscoring that hemostasis in GvHD is disturbed and dynamically regulated rather than uniformly hyperactive. Concomitant immunosuppressive and supportive treatments may also influence circulating coagulation parameters and cannot be excluded as confounders in this observational study; longitudinal studies incorporating treatment exposure and serial coagulation assessment will be needed to disentangle these mechanisms.

Elevated circulating IL-1β, IL-8, and TNF-α are consistent with their established role as central mediators of GvHD initiation and progression [25]. Beyond sustaining immune activation, IL-1β and TNF-α promote endothelial activation already evident in our TF and adhesion-molecule data, while IL-8 further amplifies neutrophil activation and NET formation. The marked increase in MCP-1 suggests enhanced monocyte recruitment and tissue infiltration [26], and elevated circulating P-selectin reflects combined platelet and endothelial activation—supporting cytokine-driven amplification as a shared upstream driver of the cellular changes described above [27].

**Scheme 1:**
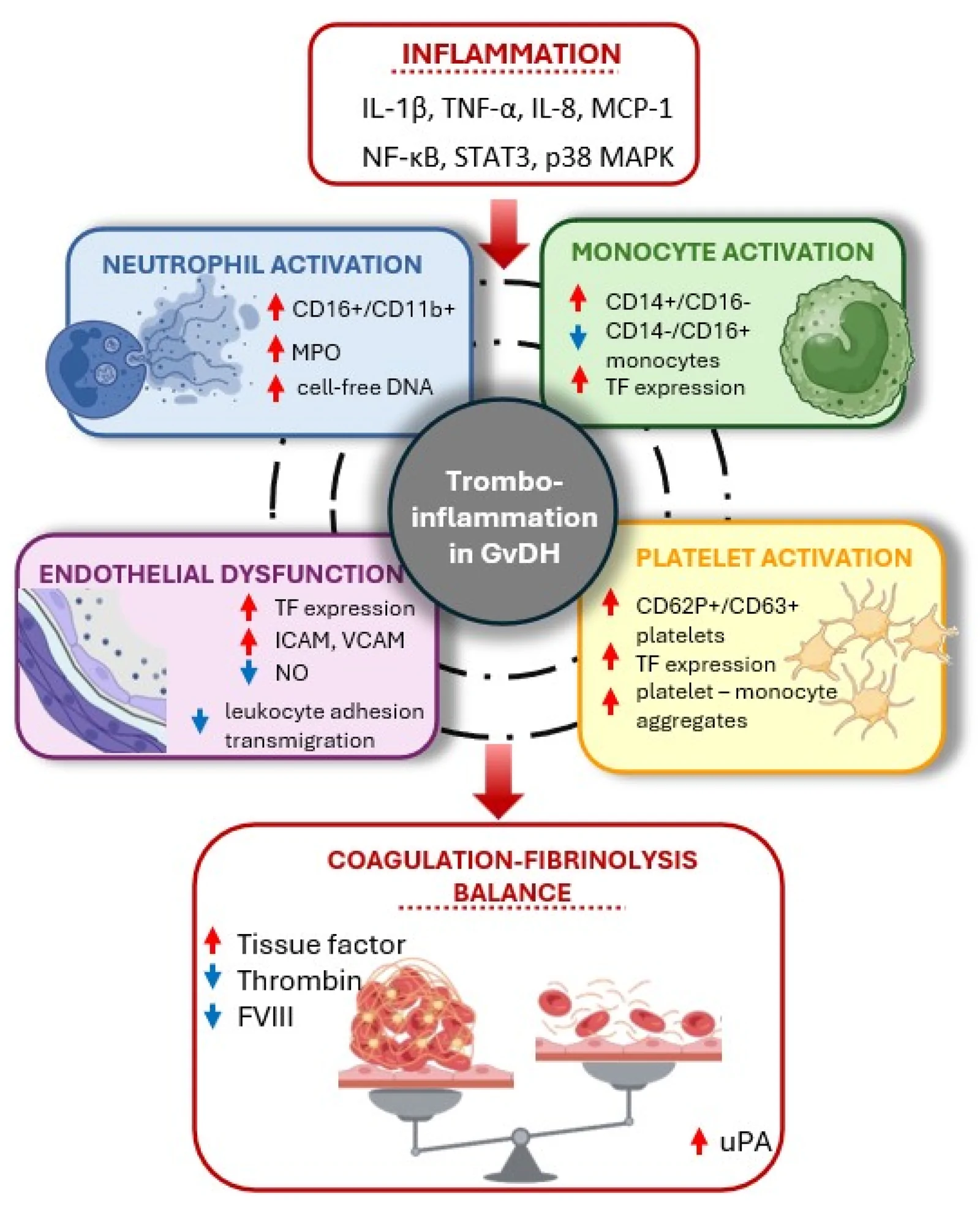
Summary model of thromboinflammation in GvHD.

Activation of the platelet–endothelial–monocyte axis provides further mechanistic support for this thromboinflammatory phenotype. Activated platelets are increasingly recognized as immune modulators that amplify inflammation through reciprocal leukocyte and endothelial interactions, in addition to their hemostatic role [28]. Increased frequencies of TF-positive platelet subsets, together with enhanced CD62P and CD63 expression, indicate a pronounced procoagulant platelet phenotype, while the marked expansion of platelet–monocyte aggregates highlights cellular crosstalk that further amplifies TF expression, cytokine production, and endothelial adhesion [29]. Together with the endothelial CD31^+^/CD142^+^ expansion, this axis represents the point at which thromboinflammation and vascular dysfunction converge (Scheme 1).

Hypoxia further amplified this inflammatory phenotype: mononuclear cells and granulocytes from patients with GvHD showed greater HIF-1α induction following TNF-α stimulation under both normoxic and hypoxic conditions, together with increased ICAM-1 and VCAM-1 expression under hypoxic conditions. As a central regulator of cellular adaptation to hypoxia, HIF-1α promotes glycolytic reprogramming, inflammatory cytokine production, and adhesion molecule expression [12,30], and the observed synergy between hypoxia and inflammatory cytokines is likely to facilitate leukocyte recruitment and retention within target organs [31]. Together with impaired leukocyte–endothelial interactions and reduced systemic NO bioavailability, these findings support endothelial dysfunction as a central, hypoxia-sensitive feature of GvHD pathophysiology [31,32].

These cellular and cytokine changes were paralleled by coordinated activation of NF-κB, STAT3, and p38 MAPK—interconnected signaling networks integrating cytokine stimulation, cellular stress, and innate immune activation. Persistent NF-κB activation promotes transcription of proinflammatory cytokines, adhesion molecules, and coagulation-related mediators; STAT3 contributes to sustained immune activation and chronic inflammation, including in GvHD specifically [33,34,35,36]; and p38 MAPK reflects stress-responsive signaling previously implicated in the pathogenesis of GvHD [37]. Rather than acting independently, these pathways likely cooperate to sustain the endothelial dysfunction and thromboinflammatory responses described above [38].

Taken together, our findings support an integrated model in which innate immune activation, endothelial dysfunction, dysregulated fibrinolysis, platelet–leukocyte interactions, and hypoxia-responsive signaling mutually reinforce one another as a self-perpetuating thromboinflammatory circuit in GvHD. This model identifies several potential therapeutic targets beyond conventional T-cell-directed immunosuppression, including inhibition of NET formation, modulation of platelet–leukocyte interactions, suppression of TF-dependent coagulation, restoration of endothelial homeostasis, and targeting of hypoxia-responsive and kinase-dependent inflammatory pathways [38]—a multimodal strategy that may help limit vascular injury and improve outcomes in GvHD. It should be noted that this integrated model represents a mechanistically plausible interpretation of the observed inflammatory, endothelial, platelet, and hemostatic alterations rather than evidence of a direct causal sequence.

A major limitation is the relatively small sample size available for several cellular and biochemical analyses. Although the overall cohort comprised 29 patients with diagnosed GvHD, individual assays were performed in smaller, partially overlapping subsets due to sample availability and assay-specific requirements; findings from the smallest subgroups—including the monocyte TF and HIF-1α experiments—should therefore be considered exploratory and hypothesis-generating rather than definitive. Because NET-specific markers such as MPO-DNA complexes or citrullinated histone H3 were not assessed, a direct increase in NET formation cannot be conclusively established, and future studies incorporating more specific NET biomarkers are needed to validate their contribution to GvHD-associated inflammation and hemostatic dysregulation. Circulating endothelial cells were identified primarily based on CD31 expression, which is not lineage-exclusive; future studies using multi-marker panels (e.g., CD31^+^/CD146^+^/CD45^−^) would allow more definitive characterization of the circulating endothelial compartment. Nevertheless, the integrated analysis presented here provides novel insight into the interplay between innate immunity, endothelial injury, dysregulated hemostasis, and hypoxia-responsive signaling in the pathophysiology of GvHD.

## 5. Conclusions

In conclusion, our findings demonstrate that GvHD is characterized by coordinated activation of innate immune, endothelial, and coagulation pathways that collectively establish a systemic thromboinflammatory state. Enhanced NET formation, dysregulated fibrinolysis, TF expression, platelet–monocyte interactions, endothelial dysfunction, and activation of hypoxia-responsive inflammatory signaling appear to represent interconnected components of a self-perpetuating pathogenic network rather than isolated biological abnormalities. These observations expand the current understanding of GvHD pathogenesis by highlighting thromboinflammation as a central mechanism linking immune activation with vascular injury and hemostatic dysregulation. Beyond conventional T-cell-directed immunosuppression, therapeutic strategies targeting NET formation, platelet–leukocyte interactions, TF-dependent coagulation, endothelial dysfunction, or hypoxia-associated inflammatory pathways may provide complementary approaches for reducing vascular injury and improving clinical outcomes. Further mechanistic and prospective clinical studies are warranted to determine whether modulation of these thromboinflammatory pathways can improve the prevention and treatment of GvHD.

## Data Availability

The data are not publicly available due to privacy and ethical restrictions.

## Supplementary Materials

The following supporting information can be downloaded at: https://www.mdpi.com/article/10.3390/cells15181690/s1.

## Abbreviations

ALL	Acute lymphoblastic leukemia
AML	acute myeloid leukemia
APC	allophycocyanin
EFS	event-free survival
FBS	fetal bovine serum
FITC	fluorescein isothiocyanate
FVIII	plasma factor VIII
GvHD	graft-versus-host disease
HIF-1α	hypoxia-inducible factor-1 alpha
HL	Hodgkin lymphoma
HMEC-1	human microvascular endothelial cells-1
ICAM-1	intercellular adhesion molecule-1
IL-1β	interleukin-1 beta
IL-8	interleukin 8
MCP-1	monocyte chemoattractant protein-1
MDS	myelodysplastic syndrome
MNCs	mononuclear cells
MPO	myeloperoxidase
NETs	neutrophil extracellular traps
NF-κB	nuclear factor kappa B
NHL	non-Hodgkin lymphoma
NO	nitric oxide
p38 MAPK	p38 mitogen-activated protein kinase
PE	phycoerythrin
PerCP/Cy5.5	peridinin chlorophyll protein complex/cyanine 5.5
PI	potassium iodide
pNA	para-nitroaniline
STAT3	signal transducer and activator of transcription 3
TF	tissue factor
TNF-α	tumor necrosis factor-alpha
uPA	urokinase plasminogen activator
VCAM-1	vascular cell adhesion molecule-1
