# Supplementary material for: Activation of Inflammatory and Coagulation Pathways in Patients with Graft-Versus-Host Disease
Source: Cells. 2026 Sep 17;15(18):1690. doi: 10.3390/cells15181690 (PMC13605787; doi:10.3390/cells15181690)
Supplement: Supplementary file 1 [file cells-15-01690-s001.zip › cells-4516977-supplementary-tables.pdf]

Supplementary Table S1. Demographic and clinical characteristics of the GvHD cohorts.

Patient No.	Age	Sex	Primary disease	Thrombosis	Time HSCT to GvHD (months)	GvHD type	Grade / Severity	Organs involved	Immunosuppressive therapy at sampling
1	2	M	AML		1	Chronic	Non-severe	lungs, eyes, oral cavity	inhalatory corticosteroids
2	31	F	HL		1	Acute	Grade IV	skin, gut	corticosteroids
3	45	M	B-ALL		4	Chronic	Non-severe	skin, eyes	ruxolitinib, ECP
4	48	F	T-ALL		2	Acute	Grade I	skin	no
5	20	M	HL		1	Acute	Grade I	skin	no
6	30	F	HL		2	Chronic	Non-severe	skin	no
7	22	M	B-ALL		9	Acute	Grade III	skin	corticosteroids, tacrolimus
8	54	M	AML	VT	38	Chronic	Non-severe	skin, eyes, oral cavity	no
9	60	M	MDS-MLD		9	Acute	Grade IV	skin, gut, liver	corticosteroids, cyclosporine, budesonide
10	54	M	MDS-MLD		8	Chronic	Non-severe	skin, oral cavity	corticosteroids
11	19	M	B-ALL		12	Chronic	Severe	skin, liver	corticosteroids
12	15	M	T cell NHL lymhoblasticum	VT, AT	2	Chronic	Severe	skin, lungs, esophagus	corticosteroids
13	32	M	B-ALL	VT	2	Chronic	Severe	skin, lungs	ruxolitinib, ECP, corticosteroids
14	43	F	AML		8	Chronic	Non-severe	skin, liver	no
15	55	M	AML		36	Acute	Grade II	skin, liver	corticosteroids
16	46	F	MDS		3	Acute	Grade III	skin	corticosteroids
17	52	F	MF		2	Acute	Grade III	skin, liver	corticosteroids, MMF, ECP
18	45	F	B-ALL		13	Chronic	Severe	skin, lungs, gut	ruxolitinib, ECP, corticosteroids
19	28	F	HL		13	Acute	Grade IV	gut	no
20	37	M	HL	VT	7	Chronic	Non-severe	skin	no
21	25	F	B-ALL		2	Acute	Grade III	skin	corticosteroids
22	37	M	AML	AT	1	Chronic	Severe	skin, upper gut, liver, eyes	ruxolitinib, ECP, cyclosporine
23	20	F	AA		3	Acute	Grade III	skin, gut, liver	corticosteroids, budesonide
25	20	M	B-ALL		7	Acute	Grade II	skin	no
25	62	M	AML		3	Chronic	Severe	skin, eyes	ECP
26	25	F	B-ALL		3	Acute	Grade I	skin	no
27	37	M	AML		1	Acute	Grade I	skin	corticosteroids
28	25	M	B-ALL		4	Acute	Grade III	skin, gut	corticosteroids
29	60	F	AML		8	Chronic	Non-severe	skin	corticosteroids

VT – venous thrombosis, AT – arterial thrombosis

Supplementary Table S2. Details of antibodies used (target, clone, fluorophore, manufacturer, dilution) for FACS and Western Blot.

**Antibody**	**Clone**	**Fluorophore**	**Dilution**	**Manufacturer**
CD16	3G8	FITC	5 μL/Test	Elabscience
CD142 (Tissue Factor)	NY2	APC	1:50	BioLegend
CD14	M5E2	PE	5 μL/Test	Elabscience
CD31	WM59	PE	1:50	BioLegend
CD62P	AK4	FITC	1:50	BioLegend
CD63	H5C6	PE	1:50	BioLegend
CD61	HIP11	PE	5 μL/Test	Elabscience
CD11b	M1/70	PE	5 μL/Test	Elabscience
CD45	H130	FITC	1:50	BioLegend
CD41	HIP8	PerCP/Cy5.5	1:50	BioLegend
phospho-NF-κB (Ser536, WB)	Polyclonal	—	1:1000	Cell Signaling Technology
total NF-κB (WB)	Polyclonal	—	1:1000	Elabscience
phospho-p38 (Tyr180/Tyr182, WB)	Polyclonal	—	1:1000	Elabscience
total p38 (WB)	Polyclonal	—	1:1000	Elabscience
STAT3 (WB)	124H6	—	1:1000	Cell Signaling Technology
phospho-STAT3 (Tyr705, WB)	Polyclonal	—	1:1000	Cell Signaling Technology
